# Supplementary figures and images for: Renal hyperfiltration as a risk factor for chronic kidney disease: A health checkup cohort study
Source: PLoS One. 2020 Sep 3;15(9):e0238177. doi: 10.1371/journal.pone.0238177 (PMC7470278; doi:10.1371/journal.pone.0238177)

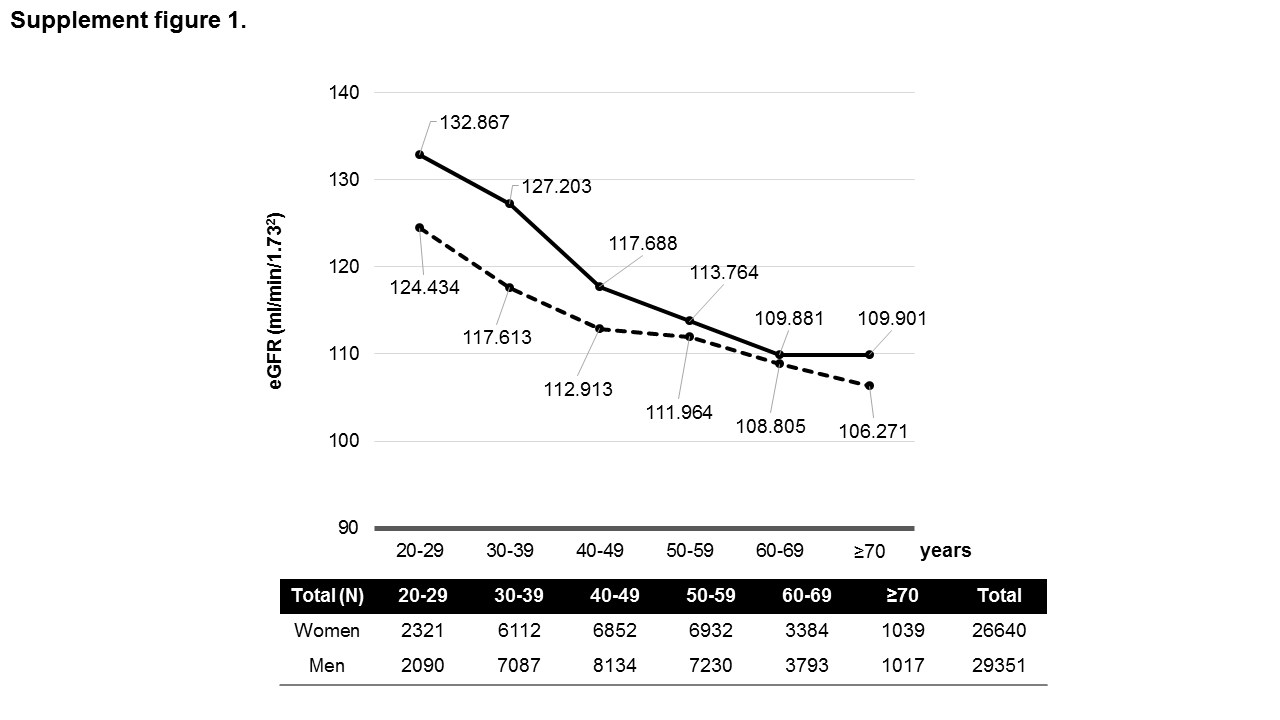

Supplement: S1 Fig — The 97.5th percentiles are shown in 10-year age groups. RHF was defined as an estimated glomerular filtration rate over the age- and sex-specific 97.5th percentile. (TIF) [file pone.0238177.s001.TIF]
