# Supplementary material for: Renal hyperfiltration as a risk factor for chronic kidney disease: A health checkup cohort study
Source: PLoS One. 2020 Sep 3;15(9):e0238177. doi: 10.1371/journal.pone.0238177 (PMC7470278; doi:10.1371/journal.pone.0238177)
Supplement: S1 Table — (DOCX) [file pone.0238177.s002.docx]

**S1 Table. The comparison of body composition between RHF and no RHF group according to body mass index (BMI)**

| BMI (kg/m2) |  | No RHF | RHF | P |
| --- | --- | --- | --- | --- |
| <20 |  | N=2568 | N=130 |  |
|  | Lean body mass (kg) | 35.8±5.1 | 34.6±5.5 | 0.024 |
|  | Body fat mass (kg) | 11.7±2.6 | 11.8±2.8 | 0.830 |
|  | Lean body mass percent (%) | 71.2±5.0 | 70.5±5.6 | 0.163 |
|  | Body fat percent (%) | 23.6±5.2 | 24.3±6.0 | 0.192 |
| 20-24 |  | N=10812 | N=467 |  |
|  | Lean body mass (kg) | 43.1±8.0 | 41.7±7.7 | <0.001 |
|  | Body fat mass (kg) | 16.0±3.3 | 16.0±3.4 | 0.826 |
|  | Lean body mass percent (%) | 69.0±5.8 | 68.4±6.0 | 0.034 |
|  | Body fat percent (%) | 26.1±6.1 | 26.8±6.2 | 0.032 |
| 25-29 |  | N=5941 | N=245 |  |
|  | Lean body mass (kg) | 49.8±8.8 | 46.6±8.8 | <0.001 |
|  | Body fat mass (kg) | 21.9±4.0 | 22.6±3.8 | 0.004 |
|  | Lean body mass percent (%) | 65.9±5.8 | 63.9±5.6 | <0.001 |
|  | Body fat percent (%) | 29.5±6.1 | 31.6±5.9 | <0.001 |
| ≥30 |  | N=1029 | N=43 |  |
|  | Lean body mass (kg) | 54.7±10.4 | 50.9±9.9 | 0.017 |
|  | Body fat mass (kg) | 33.2±6.7 | 35.2±7.7 | 0.097 |
|  | Lean body mass percent (%) | 59.5±6.0 | 56.6±6.6 | 0.009 |
|  | Body fat percent (%) | 36.5±6.4 | 39.4±7.0 | 0.009 |

*Body fat and lean body mass were measured using bioelectrical impedance analysis in 21,238 patients.
