# Supplementary material for: Renal hyperfiltration as a risk factor for chronic kidney disease: A health checkup cohort study
Source: PLoS One. 2020 Sep 3;15(9):e0238177. doi: 10.1371/journal.pone.0238177 (PMC7470278; doi:10.1371/journal.pone.0238177)
Supplement: S2 Table — (DOCX) [file pone.0238177.s003.docx]

**S2 Table. Risks for the development of 30% decline in eGFR by monocyte tertiles and renal hyperfiltration (RHF) in participants without diabetes, high SBP, and proteinuria**

|  | No RHF | RHF |
| --- | --- | --- |
| Monocyte tertiles | HR (95% CI) | HR (95% CI) |
| 1st | 1.00 | 2.868 (0.769-10.692) |
| 2^nd^ | 1.168 (0.529-2.580) | 9.110 (2.918-28.447) |
| 3^rd^ | 1.265 (0.561-2.855) | 5.336 (1.551-18.358) |

Risk are adjusted by age, sex, systolic blood pressure, body mass index, estimated glomerular filtration rate, fasting glucose, hemoglobin, aspartate transaminase, aspartate aminotransferase, alkaline phosphatase, total cholesterol, diabetes, hypertension, coronary artery disease, and malignancy.
