# Supplementary material for: Renal hyperfiltration as a risk factor for chronic kidney disease: A health checkup cohort study
Source: PLoS One. 2020 Sep 3;15(9):e0238177. doi: 10.1371/journal.pone.0238177 (PMC7470278; doi:10.1371/journal.pone.0238177)
Supplement: Attachment — Submitted filename: response_reveiwers_0809.docx [file pone.0238177.s005.docx]

**Dear reviewers**

Thank you for your thoughtful comments regarding our manuscript. We took your comments seriously and revised our manuscript again.

Reviewer #1: The previous reviewer's comments have been adequately addressed and improvements in content have been recognized.

(1) Figure 1 attached to the manuscript, which is divided into Figure 1 A-D, is described in the text as Figure 2 A-D, and there is no Figure 1 appended to the "Materials and Methods, Participants" section.

Thank you for your correction.

(2) Similarly, Figure 2, which is attached to the manuscript, is shown as Figure 3 in the text (page 10, line 7).

Thank you for your correction.

(3) Figure legends is similarly described as Figure 1 and 2, which is not consistent with Figure 1-3 in the manuscript.

Thank you for your correction.

From the text, it looks like a new Figure 1 has been added, and if so, I'd like to check that Figure.

Reviewer #2: You answered all the questions asked at the first submission and corrected the manuscript with proper supplemental information.

Thank you for your consideration.

Sincerely,

**Sang Kyung Jo, MD, PhD**

Professor

Division of Nephrology, Department of Internal Medicine, Korea University Medical College

Address : Korea University Anam Hospital, Koreadae-Ro 73, Sungbuk-Gu

Seoul, Republic of Korea 02841

e-mail : [sang-kyung@korea.ac.kr](mailto:sang-kyung@korea.ac.kr)
